# The Alterations of IL-1Beta, IL-6, and TGF-Beta Levels in Hippocampal CA3 Region of Chronic Restraint Stress Rats after Electroacupuncture (EA) Pretreatment

**DOI:** 10.1155/2014/369158

**Published:** 2014-03-25

**Authors:** Tianwei Guo, Zhuo Guo, Xinjing Yang, Lan Sun, Sihan Wang, A. Yingge, Xiaotian He, Tu Ya

**Affiliations:** ^1^School of Acupuncture-Moxibustion and Tui Na, Beijing University of Chinese Medicine, Beijing 100029, China; ^2^School of Basic Medical Science, Inner Mongolia Medical University, Hohhot 010110, China; ^3^Department of Tradition Chinese Medicine, Sanlitun Health Service Center, Beijing 100027, China

## Abstract

Immunological reactions induced by proinflammatory cytokines have been involved in the pathogenesis of depressive disorders. Recent studies showed that Electroacupuncture (EA) was able to reduce depressive symptoms; however, the underlying mechanism and its potential targets remain unknown. In the present study, we used a 21-day chronic restraint stress rats as a model to investigate how EA could alleviate depression. Open field test was carried out to evaluate the depressive symptoms at selected time points. At the end of study, immunohistochemistry (IHC) was performed to detect the expressions of IL-1beta, IL-6, and TGF-beta in hippocampal CA3 region. We found that chronic restraint stress significantly decreased behavioral activities, whereas EA stimulation at points Baihui (GV 20) and Yintang (GV 29) showed protective effect during the test period. In addition, the IL-1beta, IL-6, and TGF-beta increased in rats exposed to chronic restraint stress, while EA downregulated the levels of IL-1beta and IL-6. These findings implied that EA pretreatment could alleviate depression through modulating IL-1beta and IL-6 expression levels in hippocampal CA3 region.

## 1. Introduction

Depression, with a lifetime prevalence of up to 17%, is the leading cause of disability and ranks the 4th among diseases contributing to the global burden [1]. Various medicine treatments including antidepressant medications and psychology therapies play a pivotal role in depression treatment; however, almost one-fourth of patients are unable to achieve favorable effects, especially the improvement of somatic symptoms [2]. Thus, seeking an alternative therapy for depression is an urgent issue which is needed to be addressed. Study has shown that prevention at early stage appears to be the best option to minimize the progression of depression [3]. In clinical practice, electronic acupuncture (EA) has been proved to be an effective therapy in treating mental disorders. Studies have shown that EA can mitigate depression as shown in reduced Hamilton Depression Rating Scale scores in treated patients. In comparison with antidepressants, EA presented comparable therapeutic effects but with faster onset of action and better response rate [4, 5]. Although EA showed promising effects in alleviating the progression of depression, the underlying mechanism is poorly understood.

Over the past decades, large bodies of evidences have suggested that major depression is linked with sign of immunological activation. Specifically, activation of the inflammatory response system (IRS), such as increased production of proinflammatory cytokines, is considered to be the key factor for depression [6]. Both clinical and experimental studies indicated that increased concentration of certain types of cytokine may serve as a leading cause of stress and depression [7]. Dowlati et al. showed that high levels of IL-6 and TNF-alpha were found in depressed patients compared with control subjects [8]. Abbasi showed that antidepressant celecoxib can reduce HDRS scores as well as IL-6 concentration in patients with major depressive disorders. In animal study, chronic stress-induced depressive mice showed an increased IL-1 level in brain tissue [9]. Hippocampus, as a part of limbic system, plays an important role in the emotion regulation. Repeated stress causes atrophy of dendrites in hippocampal CA3 region [10]. In addition, CA3 neurons are more vulnerable to damages compared with dentate granule and CA1 neurons [11]. Therefore, hippocampal CA3 region is a crucial part for observing the physical changes during chronic stress.

Recent studies implied EA might function via modulation of nerve-endocrine-immune network [12]. The pathogenesis of depressive symptoms is characterized as a complex of network dysfunction in which factors including neurotransmitters, hormones, and cytokines, interact intimately. Therefore, a research strategy focusing on nerve-endocrine-immune network might be applied to identify the key player which involved in EA treatment in depression. In the present study, we hypothesized that EA could modulate proinflammatory cytokine levels and thus reduced depression syndrome.

## 2. Material and Methods

### 2.1. Animals

A total of 30 specific pathogen-free (SPF) Sprague Dawley rats (260~280 g) were supplied by the Institute of Laboratory Animal Sciences, China Academy of Medical Science, animal license number SCXF (Jing)2009-0017. Animals were housed at (22 ± 2)°C, 45% humidity, in 12-hour light/dark cycles (light on at 8:00 am), with free access to food and water. The study was performed 3 days after environment acclimations of the rats. The protocols were conducted in compliance with the Guidance Suggestions for the Care and Use of Laboratory Animals formulated by the National Institute of Health, as well as the 3R principle: Reduction, Replacement, and Refinement. All experiment procedures were approved by the Animal Care and Use Committee at Beijing University of Chinese Medicine.

### 2.2. Groups and Treatment

For control group, no model induction and treatment were performed. For model group, chronic stress was conducted for 21 days on a daily basis with method described as follows: rats were restrained with self-made cylinder-shaped wire net (20 cm in length and 5 cm in diameter) from 9 am to 3 pm. After restraints, they were released for free access to water and food. For EA group, EA pretreatment was conducted daily prior to restraint for 21 days, restraint method was the same as model group.

### 2.3. EA Pretreatment

During acupuncture administration, rats were maintained within a cloth bag. Two points were selected: Baihui (GV20) and Yintang (GV29). GV20 is located above the apex auriculate, on the midline of the head. GV29 is located at the middle point between two eyes [13]. Sterilized disposable stainless steel needles (0.20∗25 mm, Hua Tuo brand, manufactured by Suzhou medicine Co., Ltd., Suzhou, Jiangsu, China) were inserted obliquely as deep as 3–5 mm for both points. Following the insertions, electrodes were added to the handle of the needles (electric acupuncture apparatus used: Hans-100 A, manufactured by Nanjing Jisheng medicine science Co., Ltd., Nanjing, Jiangsu, China). Electricity simulation parameters were 1 mA, 2 Hz, for 20 minutes.

### 2.4. Open Field Test

At selected time points: day 0, day 7, day 14, and day 21, the open field test was conducted with modifications of previous studies [14]. The apparatus, wood in material, was comprised of a square arena 80 × 80 cm with 40 cm high wall. It was divided into 25 × 25 equal squares which had been drawn in the floor of the arena. A single rat was gently placed in the center of the floor in order to explore the arena for 3 min. The activity of the rat was recorded by a camera installed on top of the lateral high wall. Two observers, blind to the experiment, counted the crossing numbers (defined as at least three paws in a square) and the rearing numbers (defined as the rat standing upright on its hind legs) from a monitor connected to the camera which was set one meter away from the apparatus. After one rat finished the test, alcohol was applied to clean the floor to exclude the intervention of odor signals. The body weight was measured on day 0, day 7, day 14, and day 21 of the experiment.

### 2.5. Frozen Section and Immunohistochemistry

At the end of the study, rats were deeply anesthetized with 10% chloral hydrate (0.3 mL/100 g, i.p.) and perfused with 4°C 4% paraformaldehyde from left apex. After perfusion, hippocampus was harvested and embedded in liquid nitrogen. For sections preparation, chiasma opticum was positioned at first; then the tissue was cut into 20 *μ*m coronal sections till 2-3 mm posterior to chiasma opticum on a sliding microtome (Leica CM1850, German) and stored at −20°C. Immunohistochemistry was carried on as previously described [15]. The primary antibodies (goat against rat IL-6 IgG, product number: SC-1265R; rabbit against rat IL-1beta IgG, product number: SC-7884; rabbit against rat TGF-beta IgG, product number: SC-146 manufactured by Santa Cruz Biotechnology (Shanghai) Co., Ltd., Shanghai, China) were added and then incubated over night at 4°C. Secondary antibodies conjugated with horseradish peroxidase (HRP) was added afterward and incubated at 37°C for 1 hour. HRP substrates were applied at last for color development. The protein expressions were quantified by Integral Optical Density (IOD) using Image Pro Plus 6.0 software. For each rat, 3 to 4 sections were applied and mean value was obtained to determine the expression level.

### 2.6. Statistical Analysis

Data were presented as means ± S.E.M. SPSS 20.0 (SPSS Inc, Chicago, USA) was deployed for data analysis with one-way ANOVA method after the test of normal distribution and homogeneity of variance, followed by post hoc multiple comparison. Statistical significance was set to *P* < 0.05, while highly statistical significance was set to *P* < 0.01.

## 3. Results

### 3.1. Effects of EA Pretreatment on Body Weight

**Figure 1:**
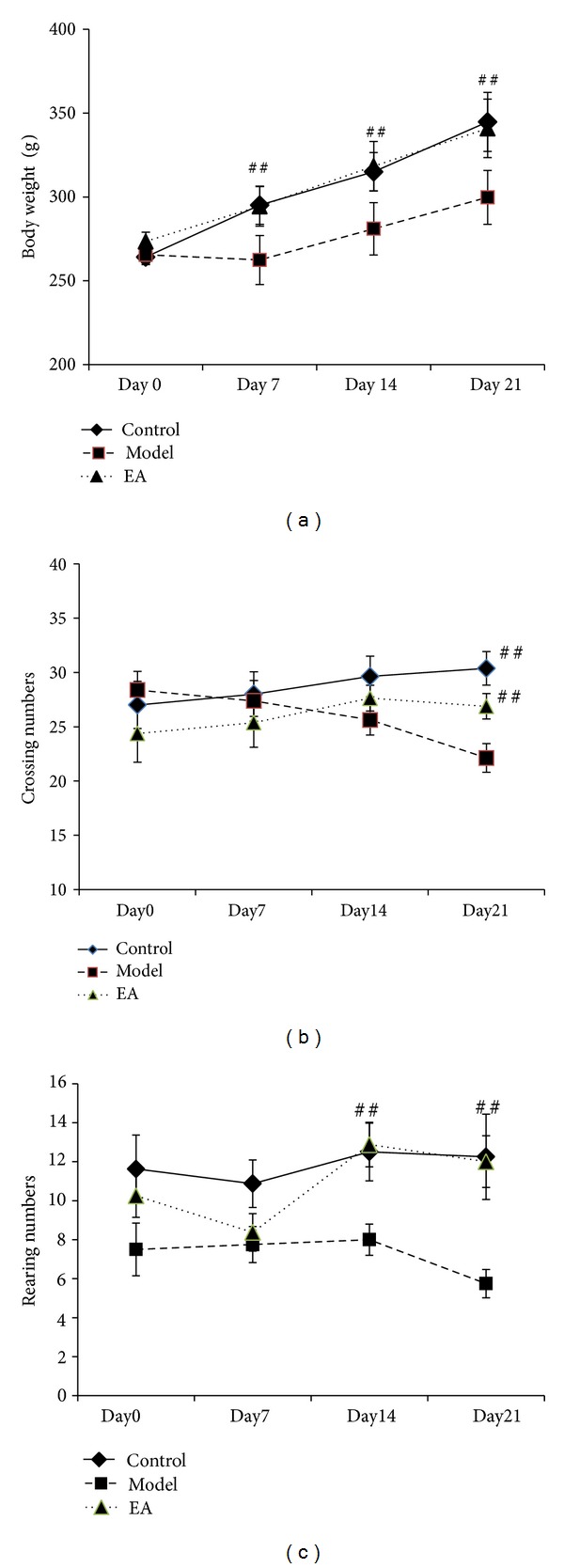
The effect of electric acupuncture (EA) on body weight and locomotor activity in open field test at selected time points in the following groups (*n* = 8 per group): control, model, and EA. (a) Body weight. (b) Crossing numbers in open field test. (c) Rearing numbers in open field test. ^##^ *P* < 0.01 as compared with model group.

As shown in Figure 1(a), the body weight increased slowly in model group in contrast with those in control and EA group. 21 days after induction, the body weights in control group and EA group were significantly higher compared with that in model group (*P* < 0.01), whereas no significant differences was found between control group and EA group. This result suggested that EA has a protective effect on body weight.

### 3.2. Effects of EA Pretreatment on Open Field Test

We used the open field test to evaluate the exploratory and locomotor activity [16, 17]. As seen in Figure 1(b), model group presented a decline tendency in crossing numbers during the 21-day restraint stress procedure, while control and EA group showed a rise tendency. 21 days after induction of the crossing numbers in model group was significantly decreased in comparison with those in control and EA group with statistically significant differences (*P* < 0.01). There was no difference between control and model group (*P* > 0.05). In addition, restraint stress stimuli remarkably reduced the rearing numbers in model group compared with that in control and EA group 21 days after induction, whereas no significant difference was found between control and EA group. The results indicated that EA plays a crucial role in ameliorating stress-impaired exploratory and locomotor activities.

### 3.3. Effects of EA Pretreatment on IL-1Beta

**Figure 2:**
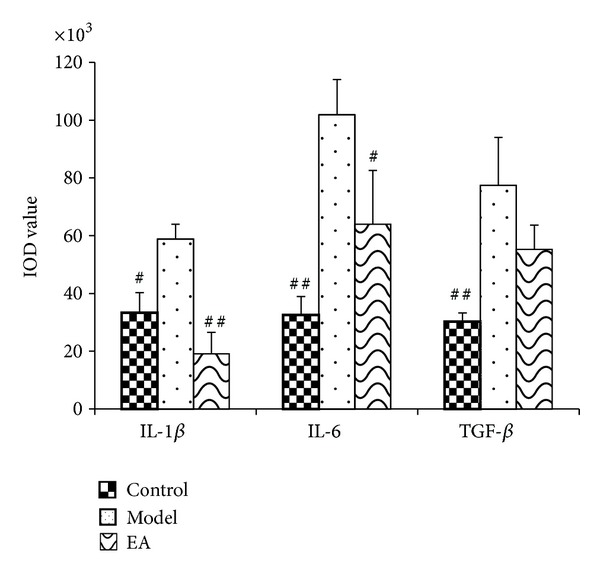
The effect of EA on IL-1beta, IL-6, and TGF-beta protein expression in hippocampus (HP) CA3 region in the following groups (*n* = 8 per group): control, model, and EA. ^##^ *P* < 0.01, ^#^ *P* < 0.05 as compared with model group.

**Figure 3:**
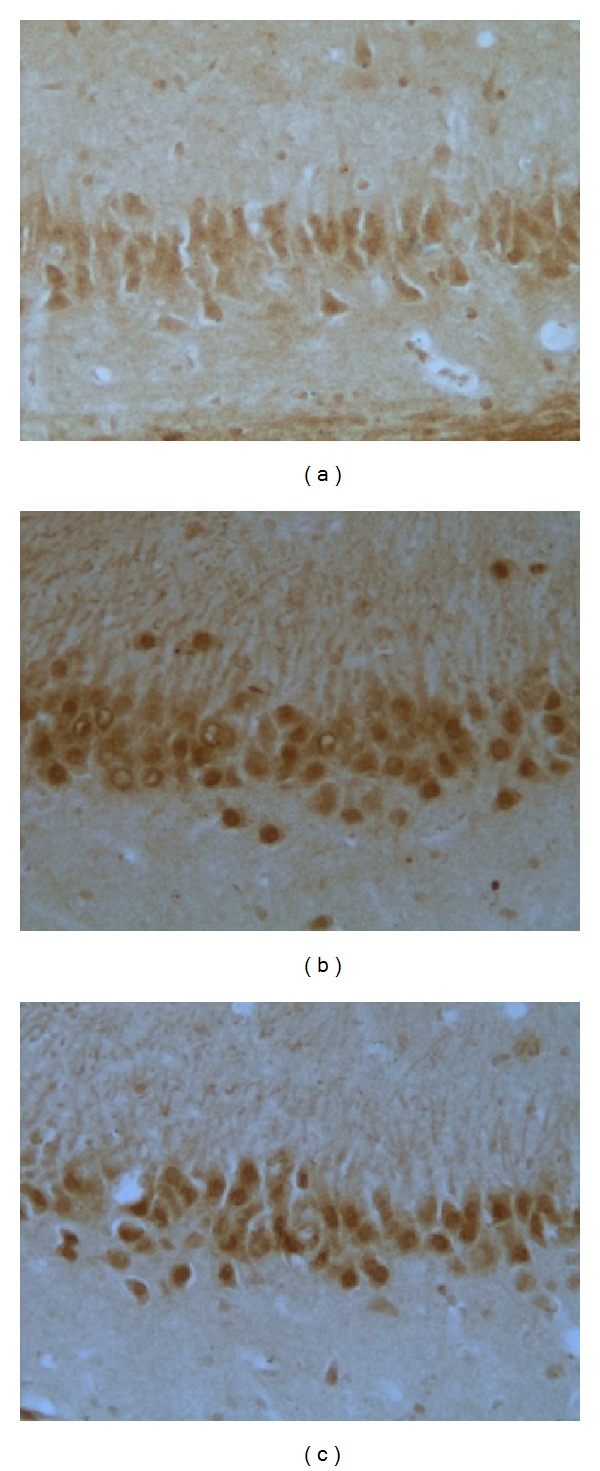
Representative immunohistochemistry results showing IL-1beta levels and neuron morphology in the hippocampus CA3 region in the following groups (*n* = 10 per group): (a) control, (b) model, and (c) EA.

As shown in Figure 2, the expression of IL-1beta in model group was significantly increased compared with that in control group (a) and EA group based on IOD value (*P* < 0.01), whereas there was no significantly difference between EA group and control group (*P* > 0.05). Figures 3(a) and 3(c) immunostaining-positive cells in hippocampal CA3 region in control and EA group were arranged in line with less cytoplasm coloring, whereas Figure 3(b) showed that in model group, large amount of positive cells, in a mass, has deep cytoplasm coloring. These results indicated that EA decreased the expression of IL1beta and showed a protective effect in hippocampal tissue.

### 3.4. Effects of EA Pretreatment on IL-6

Figure 2 showed that after a 21-day procedure, IOD value of IL-6 in model group was significantly increased compared with that in control group (*P* < 0.01) and EA group (*P* < 0.05).

**Figure 4:**
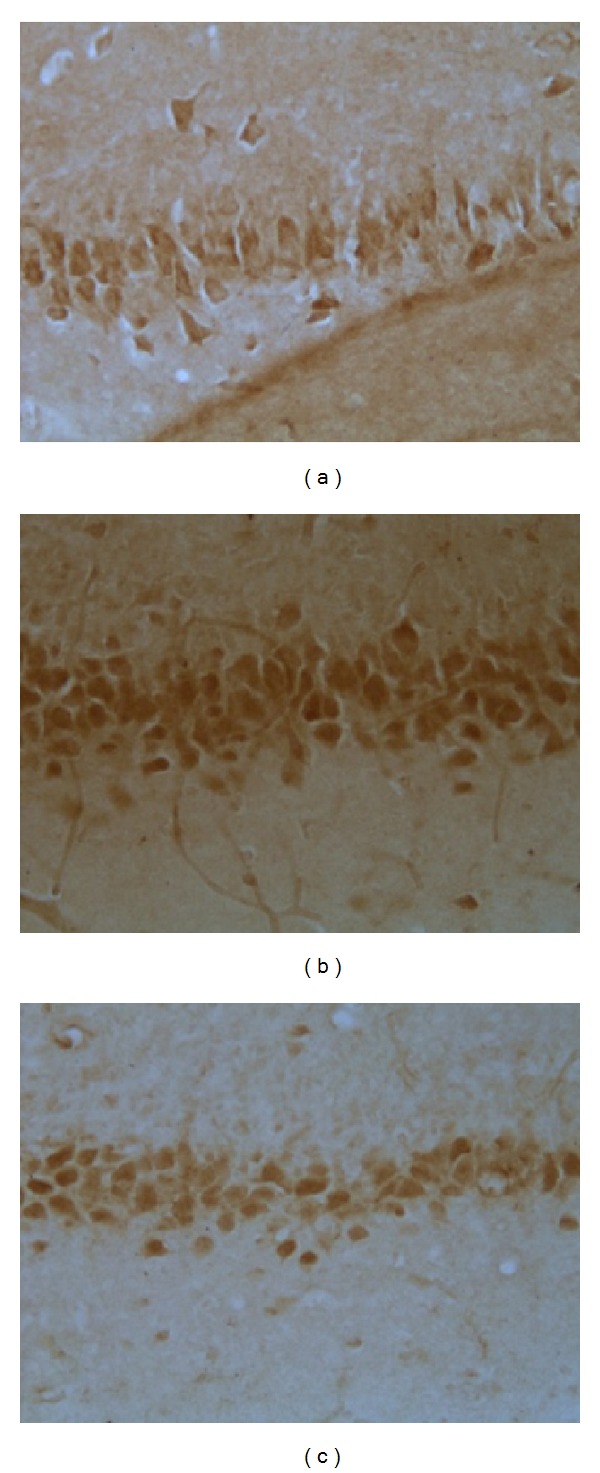
Representative immunohistochemistry results showing IL-6 levels and neuron morphology in the hippocampus CA3 region in the following groups (*n* = 8 per group): (a) control, (b) model, and (c) EA.

As shown in Figures 4(a) and 4(c) immunostaining-positive cells in hippocampal CA3 region of rats in control and EA group were arranged in line with less cytoplasm coloring.  Figure 4(b) showed that in model group, large amount of positive cells, in a mass, has deep cytoplasm coloring. The results suggested that EA suppressed the hypersecretion of IL-6 and therefore protected hippocampus against proinflammatory stress.

### 3.5. Effects of EA Pretreatment on TGF-Beta

**Figure 5:**
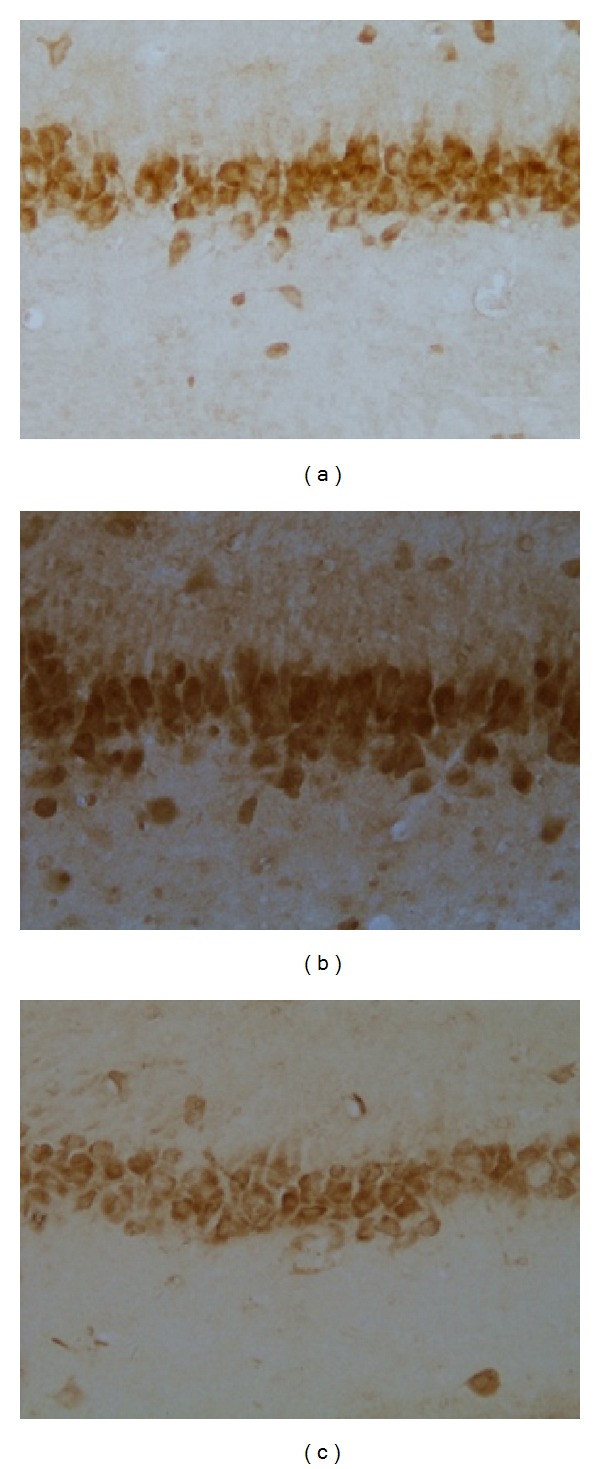
Representative immunohistochemistry results showing TGF-beta levels and neuron morphology in the hippocampus CA3 region in the following groups (*n* = 8 per group): (a) control, (b) model, and (c) EA.

Figures 5(a) and 5(c) showed that positive cells in control and EA group were arranged in line with less cytoplasm coloring, while Figure 5(b) showed that in model group, numerous positive cells distributed densely with deep cytoplasm coloring. However, as shown in Figure 2, the IOD value of TGF-beta in model group was significantly increased compared with that in control group, indicating that restraint stress can stimulate the expression of TGF-beta, whereas EA reduced its expression, but the effect was not of statistical significance.

## 4. Discussions

The major finding of the present study is that EA pretreatment modulated the expression of IL-6 and IL-1beta in hippocampal CA3 region in chronic restraint stress rats.

IL-1beta and IL-6 have been intensively investigated for their roles in depressive symptoms. Previous studies indicated that overexpression of IL-6 promotes depressive-like behavior [18, 19]. Lenczowski et al. demonstrated that IL-6 can reduce social investigatory and behavior and locomotor activity in the presence of IL-1beta. Nevertheless, controversial studies offered opposite notions that IL-6 administration failed to elicit sickness behavior [20]. The discrepancy might be explained from differences in stress category, duration, and other experimental procedures.

In parallel to most previous results, we found that elevated secretions of IL-1beta, IL-6, and TGF-beta occurred concomitantly with depressive symptoms, suggesting a hyperactivity of immune function caused by restraint stressor. Cytokines and their receptors such as IL-1, IL-2, IL-6, and TNF-*α* and some other growth factors are localized in rodent brain with highest densities in the hippocampus and hypothalamus [21, 22]. Therefore, cytokine hyperactivity can stimulate various chain reactions to harm regions related to emotion perception and regulation. The literature suggested that chronic inflammations, shown as overexpression of cytokines, can activate the enzyme degrading tryptophan which leads serotonin depletion and antioxidant defenses impairment [23–25]. In addition, cell-mediated immune cytokines can increase the synthesis of neurotoxic tryptophan catabolites (TRYCATs) which contributes to oxidative stress, impaired mitochondrial metabolism, and apoptosis.

EA has been proved to be capable in reversing excitotoxicity and apoptosis [26]. EA showed protective effects on hippocampal CA3 regions including decreasing presynaptic glutamate synthesis and release, blocking postsynaptic excitatory amino acid receptors, and terminating pathological chain reaction caused by excessive excitatory receptors to inhibit glutamate release. The essential target may be NMDA receptor which can be inhibited to decrease calcium ions influx [27]. In addition, Liang et al. showed that EA can achieve curative effects by involving in the signal pathway of Ras-MKK-JNK; specifically, EA can alleviate apoptosis by decreasing the level of Capase-3 and increasing the ratio of Bcl2 to Bad [28]. In the present study, we found significantly decreased secretion of IL-6 and IL-1beta in hippocampal CA3 region of EA pretreated stressed rats, which might indicate the target cytokines of EA in regulating immune system.

Moreover, IL-6 has been suggested to be associated with brain-derived neurotrophic factor (BDNF) which is highly involved in the physiopathology of depression [29]. It has been suggested that IL-6 activated protein kinase B and then it can phosphorylate the nuclear localization signal on DNA methyltransferase-1 (DNMT1) which hypermethylates BDNF promoter and further reduces BDNF level [30, 31]. BDNF plays a pivotal role in spine formation and synapse plasticity which facilitates the connectivity between different brain regions in limbic system. Similarly, it has also been suggested that EA can boost BDNF level through the modulation of Ras-MAPK-ERK pathway to mitigate the phosphorylation of ERK1/2 [32]. Therefore it is speculated that IL-6 serves as a mediator between EA pretreatment and its beneficial effects, specifically, EA could downregulate the expression of IL-6 to restore the level of BDNF.

IL-1beta has been shown to function synergistically with IL-6 to activate HPA axis to reduce social investigatory behavior and locomotor activity [20], indicating the underlying combined mechanism of IL-1beta and IL-6 in depression. Evidence from another acupuncture treatment research for chronic stress model indicated that action of acupuncture may be mediated by an inhibition of HPA axis via attenuated c-fos which symbolizes decreased arginine vasopressin (AVP) and corticotropin releasing hormone (CRH). Based on observed reduced expression of IL-6 and IL-1beta after EA, we think that EA suppress these proinflammatory cytokines to downregulate HPA axis hyperactivity.

TGF-beta, distinguished itself from the above cytokines with a special pathway, performs many cellular functions, including the control of cell growth, proliferation, differentiation, and apoptosis [33]. The literatures reported that TGF-beta is involved in neurodegenerative diseases such as Alzheimer's disease [34], but the correlations between TGF-beta and depression is poorly known. However, based on its pathway in physiopathology, several potential links could be found between TGF-beta and depressive disorders. The activation of TGF-beta can activate downstream MAPK pathway which has been described as to be implicated in the expression of BDNF [35]. In addition, it has been shown that TGF-beta is also involved in Ras-MKK-JNK pathway which is highly correlated to apoptosis and growth arrest, serving as an underlying mechanism of depressive symptoms. Wu demonstrated that EA can boost the expression of Bcl-2 gene to inhibit apoptosis in brain tissue after chronic stress induction [36]. Thus we hypothesized that EA may exert beneficial effects on depressive symptoms through a mechanism in which TGF-beta activating Erk1/2 pathway as well as JNK pathway. However, according to our results, TGF-beta level declined in EA group without statistical significance in comparison with model group. The role of TGF-beta in immunological activation and EA prevention warrants further investigations.

In the present study, EA pretreatment was administered under a slightly restrained condition. Our previous work (unpublished data) illustrated that no significant difference was observed in serum adrenocorticotropic hormone (ACTH) and corticosterone (CORT) level between the normal rats and normal plus acupuncture rats. It is suggested that the acupuncture administration will not induce stress response.

According to Traditional Chinese Medicine, Baihui and Yintang are points pertaining to Governor Meridian. Based on Meridian and Collateral Theory, Governor Meridian is the convergence of all the Yang meridians; therefore, stimulation on points of Governor Meridian can boost Yang qi of the whole body to reverse the pathogenesis of depression in which it is defined as yang deficiency syndrome. Meanwhile, EA pretreatment design of our study embodies one of the most critical theories in Traditional Chinese Medicine, the principle of “treating diseases prior to its onset” which attaches great significance on disease preventions.

## 5. Conclusions

In summary, the present study demonstrated that the proinflammatory cytokines IL-1beta, IL-6, and TGF-beta in rats' hippocampus mediated the onsets of depressive symptoms after chronic restraint stress inductions. Importantly, our findings suggested that EA can significantly mitigate deficit behavioral activities elicited by chronic restraint stress through a potential mechanism of immunological modulation.
